# End-tidal to arterial PCO_2_ ratio: a bedside meter of the overall gas exchanger performance

**DOI:** 10.1186/s40635-021-00377-9

**Published:** 2021-04-19

**Authors:** Matteo Bonifazi, Federica Romitti, Mattia Busana, Maria Michela Palumbo, Irene Steinberg, Simone Gattarello, Paola Palermo, Leif Saager, Konrad Meissner, Michael Quintel, Davide Chiumello, Luciano Gattinoni

**Affiliations:** 1 Department of Anesthesiology, University Medical Center Göttingen, Robert Koch Straße 40, 37075 Göttingen, Germany; 2 Department of Anesthesiology and Intensive Care, ASST Santi E Paolo Hospital, University of Milan, Milan, Italy

**Keywords:** P_ET_CO_2_, Acute respiratory distress syndrome, Severity, Monitoring

## Abstract

**Background:**

The physiological dead space is a strong indicator of severity and outcome of acute respiratory distress syndrome (ARDS). The “ideal” alveolar PCO_2_, in equilibrium with pulmonary capillary PCO_2_, is a central concept in the physiological dead space measurement. As it cannot be measured, it is surrogated by arterial PCO_2_ which, unfortunately, may be far higher than ideal alveolar PCO_2_, when the right-to-left venous admixture is present. The “ideal” alveolar PCO_2_ equals the end-tidal PCO_2_ (P_ET_CO_2_) only in absence of alveolar dead space. Therefore, in the perfect gas exchanger (alveolar dead space = 0, venous admixture = 0), the P_ET_CO_2_/PaCO_2_ is 1, as P_ET_CO_2_, P_A_CO_2_ and PaCO_2_ are equal. Our aim is to investigate if and at which extent the P_ET_CO_2_/PaCO_2_, a comprehensive meter of the “gas exchanger” performance, is related to the anatomo physiological characteristics in ARDS.

**Results:**

We retrospectively studied 200 patients with ARDS. The source was a database in which we collected since 2003 all the patients enrolled in different CT scan studies. The P_ET_CO_2_/PaCO_2_, measured at 5 cmH_2_O airway pressure, significantly decreased from mild to mild–moderate moderate–severe and severe ARDS. The overall populations was divided into four groups (~ 50 patients each) according to the quartiles of the P_ET_CO_2_/PaCO_2_ (lowest ratio, the worst = group 1, highest ratio, the best = group 4). The progressive increase P_ET_CO_2_/PaCO_2_ from quartile 1 to 4 (i.e., the progressive approach to the “perfect” gas exchanger value of 1.0) was associated with a significant decrease of non-aerated tissue, inohomogeneity index and increase of well-aerated tissue. The respiratory system elastance significantly improved from quartile 1 to 4, as well as the PaO_2_/FiO_2_ and PaCO_2_. The improvement of P_ET_CO_2_/PaCO_2_ was also associated with a significant decrease of physiological dead space and venous admixture. When PEEP was increased from 5 to 15 cmH_2_O, the greatest improvement of non-aerated tissue, PaO_2_ and venous admixture were observed in quartile 1 of P_ET_CO_2_/PaCO_2_ and the worst deterioration of dead space in quartile 4.

**Conclusion:**

The ratio P_ET_CO_2_/PaCO_2_ is highly correlated with CT scan, physiological and clinical variables. It appears as an excellent measure of the overall “gas exchanger” status.

## Introduction

The physiological dead space, which includes both the anatomical and alveolar dead space, is a strong indicator of severity and outcome of acute respiratory distress syndrome (ARDS) [1, 2]. The computation of the physiological dead space is based on the dilution of the ideal alveolar PCO_2_ (PACO_2_). This ideal PCO_2_, introduced by Riley, cannot be measured directly and it is assumed to be equal to the capillary PCO_2_ (PcCO_2_) [3] which leaves the ventilated/perfused pulmonary units [4]. As the PcCO_2_ cannot be measured directly, the arterial PCO_2_ (PaCO_2_) is assumed to be its surrogate. Therefore, the assumption on which the physiological dead space is computed is that PACO_2_, PcCO_2_ and PaCO_2_ have identical values. While this is nearly correct in the normal lung, in the diseased lung, as in ARDS, the PaCO_2_ is higher than PcCO_2_ and PACO_2_ due to the presence of venous admixture (in Riley’s model, the fraction of blood which flows through “non-aerated lung regions” maintaining the same PO_2_ and PCO_2_ of the mixed venous blood). Consequently, PaCO_2_ is the result of the weighted average of blood coming from the ideal compartment (PcCO_2_) and of mixed venous PCO_2_ (PvCO_2_) [5]. , It is therefore easy to understand why the venous admixture, i.e., a variable which measures the oxygenation impairment, has an effect on variables which describe the wasted ventilation. To compute the alveolar dead space, we may assume that the end-tidal CO_2_ (P_ET_CO_2_) is representative of the actual alveolar gases. In this case, the P_ET_CO_2_ is lower than the PACO_2_ depending on the amount of alveolar dead space.

The measurement of the P_ET_CO_2_ is easily performed in intensive care. Therefore, the alveolar dead space may be derived as follows:

$$\mathrm{Alveolar}\;\mathrm{dead}\;\mathrm{space}=1-\frac{{\mathrm{EtCO}}_2}{{\mathrm{PaCO}}_2}.$$

As the alveolar dead space, as measured by the equation above, depends both on the “true” alveolar dead space and on the extent of the venous admixture, the P_ET_CO_2_/PaCO_2_ ratio may be seen as a direct overall meter of the gas exchanger performance in a scale from 0 to 1. Indeed, a P_ET_CO_2_/PaCO_2_ ratio equal to 1 represents the perfect gas exchanger, being, in this condition, the alveolar dead space and the venous admixture equal to 0. The presence of alveolar dead space and/or venous admixture at different extent would progressively decrease this ratio from the unity, reflecting the progressive deterioration of the gas exchanger in its two components, oxygenation and CO_2_ removal.

The aim of this study is to investigate whether the P_ET_CO_2_/PaCO_2_, easily measurable at the bedside, can be an adequate tool to assess the physio-anatomical condition of the gas exchanger.

## Materials and methods

### Study population

This study population consisted of 200 patients, studied from 2003 and 2016 in two university hospitals (Policlinico Milano, Milan, Italy and University Medical Center Göttingen, Göttingen, Germany). All patients suffered from ARDS according to the Berlin criteria [6]. The ethics committee was notified and permission to use the data was granted (Göttingen Antragsnummer 14/12/12).

### Recorded variables

For each patient, the CT scans were acquired at 5, 15 and 45 cmH_2_O of airway pressure. We reported the anatomical variables derived from the CT quantitative analysis: namely, hyperinflated (− 1000/− 900 HU), well aerated (− 900/− 500 HU), poorly inflated (− 500/− 200 HU) and non-aerated (− 100/ + 100 HU) tissues [7, 8]. Recruitability was computed as the fraction of non-aerated tissue at 5 cmH_2_O minus the fraction of non-aerated tissue at 45 cmH_2_O [9]. Lung inhomogeneity was computed on a voxel-by-voxel basis, as the ratio of gas content between acinar size lung units and surrounding lung units [10]. A ratio equal to 1 would indicate perfect homogeneity, a ratio of 2 would indicate an inflation of the central lung unit double than the surrounding units. At 5 and 15 cmH_2_O of airway pressure, we collected the mechanical ventilation settings and respiratory mechanics variables (tidal volume, respiratory rate, alveolar ventilation, and respiratory system elastance), hemodynamics (systolic and diastolic arterial blood pressures, central venous pressure, heart rate, ScvO_2_ and arteriovenous O_2_ content difference) and gas exchange variables (P_ET_CO_2_, PaO_2_, PaCO_2_, PaO_2_/PaCO_2_ ratio, SaO_2_, venous admixture (*Q*_s_/*Q*_t_), physiological dead space fraction (*V*_d_/*V*_t_)). Tidal volume and FiO_2_ were kept constant at these two PEEP levels. The volumetric capnography measurements were performed with COSMO (Respironics Novametrix, Wallingford, USA).

The first analysis was done grouping the patients according to the their ARDS severity (mild, moderate–mild, moderate–severe, severe) [11]. An additional analysis was performed dividing the patients into four groups (~ 50 patients per group) based on the equal-count quartiles of their P_ET_CO_2_/PaCO_2_ ratios determined during ventilation at 5 cmH_2_O of PEEP. For details regarding the calculated variables, please refer to Additional File 1.

### Statistical methods

The normal distribution of the data was assessed by the Shapiro–Wilk test. Physiological, CT scan variables and P_ET_CO_2_/PaCO_2_ ratio were compared among groups with one-way analysis of variance or Kruskal–Wallis test as appropriate. Multiple comparisons were performed with Bonferroni correction. Two tailed, *p* values < 0.05 were considered statistically significant. These statistical analyses were performed with R (R Foundation for Statistical Computing version 3.7).

## Results

**Fig. 1:**
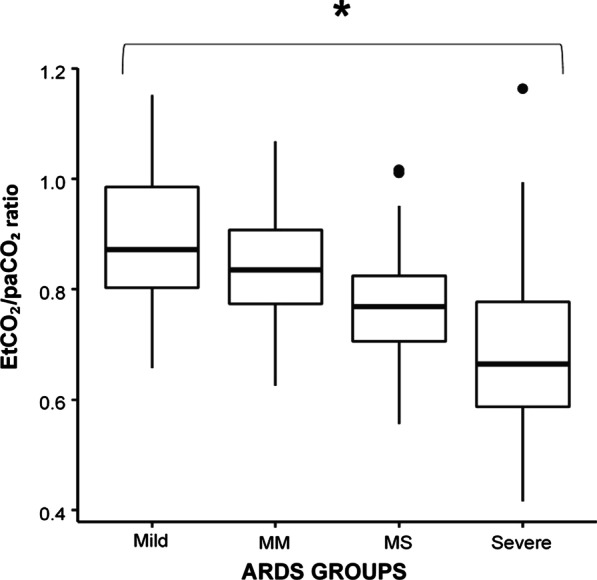
Panel A: P_ET_CO_2_/PaCO_2_ ratio among acute respiratory distress syndrome (ARDS) groups divided in Severe, Moderate–Severe (MS), Moderate–Mild (MM) and Mild groups at 5 cmH_2_O of positive end-expiratory pressure (PEEP). *p < 0.001, Mann–Whitney test

**Fig. 2:**
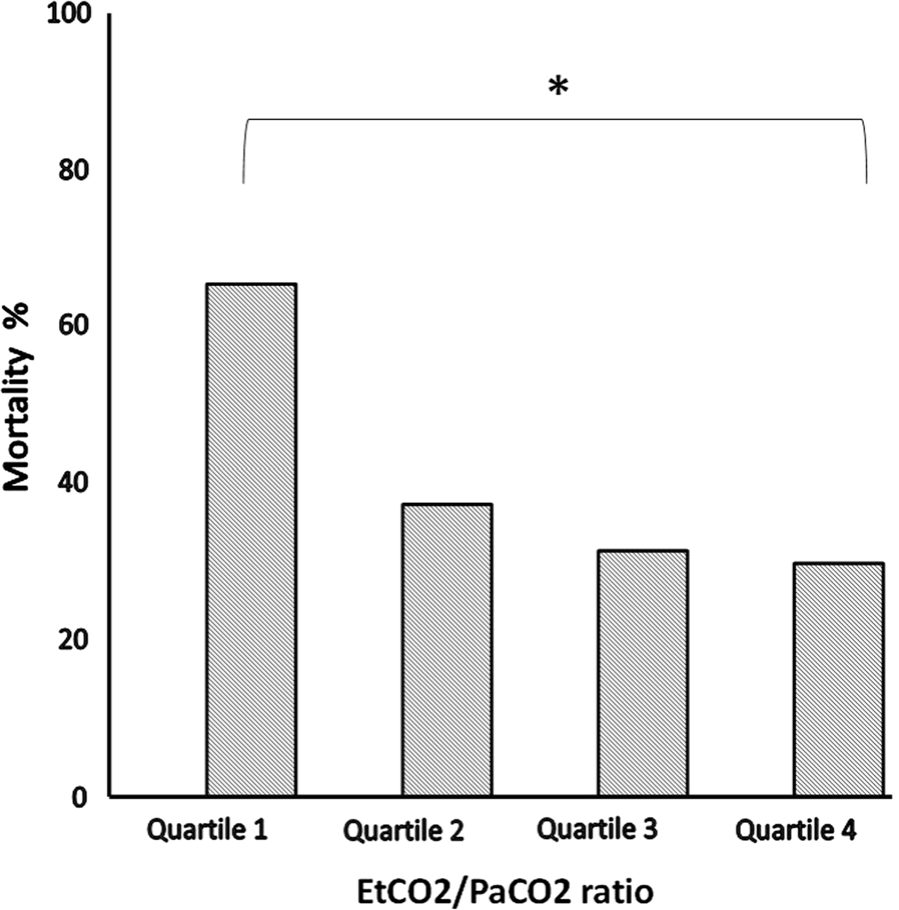
Mortality rate observed in the four quartiles of P_ET_CO_2_/PaCO_2_ ratio

The main anthropometric and the physiological characteristics of the study population obtained at 5 cmH_2_O of PEEP are presented in Table 1. Figure 1, panel A shows the P_ET_CO_2_/PaCO_2_ ratio as a function of ARDS severity. The ratio decreased linearly with increasing severity. In Fig. 2, we report the mortality rate observed in the quartiles of P_ET_CO_2_/PaCO_2_ ratio.

**Table 1:** Baseline characteristics among ARDS groups

ARDS severity	Mild	MM	MS	Severe	P
Number of patients	*N* = 33	*N* = 54	*N* = 70	*N* = 43	
Age	59.0 [43.0; 69.0]	66.0 [53.2; 76.0]	61.0 [45.2; 70.5]	63.0 [52.0; 72.5]	0.382
Sex					0.905
Female	13 (39.4)	16 (29.6)	23 (32.9)	13 (30.2)	
Male	20 (60.6)	38 (70.4)	46 (65.7)	30 (69.8)	
BMI	25.6 [22.2; 28.0]	25.0 [23.1; 27.8]	24.2 [21.9; 26.7]	26.1 [22.2; 32.0]	0.107
SAPS II	41.5 [27.8; 46.2]	40.0 [31.0; 54.2]	39.0 [33.0; 52.0]	43.0 [35.0; 54.5]	0.416
Tidal volume, ml/kg	540 [500; 600]	550 [480; 600]	502 [432; 560]	500 [428; 590]	0.082
Respiratory Rate, bpm	15.0 [12.0; 18.0]	15.0 [13.0; 18.0]	18.0 [14.0; 20.0]	18.0 [15.0; 20.0]	0.110
Plateau pressure, cmH_2_O	19.0 [16.1; 21.3]	17.0 [14.5; 20.0]	18.5 [16.3; 21.0]	18.0 [15.9; 21.0]	0.121
E_rs_, cmH_2_O/L	23.1 [17.6; 29.2]	22.3 [19.1; 25.6]	26.9 [21.4; 31.7]	26.9 [21.0; 33.2]	0.005
PaO_2_/FiO_2_ ratio	230 [218; 255]	173 [160; 183]	122 [108; 139]	79.6 [66.7; 87.4]	< 0.001
PaO_2_, mmHg	96.2 [83.0; 109]	75.2 [65.8; 81.9]	67.2 [62.0; 71.9]	60.0 [54.0; 71.3]	< 0.001
FiO_2_	0.40 [0.40; 0.45]	0.40 [0.40; 0.50]	0.55 [0.50; 0.60]	0.85 [0.70; 0.92]	< 0.001
PaCO_2_, mmHg	40.9 [38.2; 44.2]	42.9 [38.2; 49.8]	44.5 [39.7; 50.3]	52.0 [43.2; 55.0]	< 0.001
Arterial pH	7.39 [7.35; 7.46]	7.39 [7.35; 7.43]	7.38 [7.31; 7.42]	7.36 [7.30; 7.40]	0.024
ARDS causes, no. (%):					
Aspiration	1 (3.03)	5 (9.26)	7 (10.0)	2 (4.65)	
Other	5 (15.2)	5 (9.26)	11 (15.7)	5 (11.6)	
Pneumonia	8 (24.2)	21 (38.9)	35 (50.0)	28 (65.1)	
Sepsis	15 (45.5)	18 (33.3)	14 (20.0)	7 (16.3)	
Trauma	4 (12.1)	5 (9.26)	3 (4.29)	0 (0.00)	

*P* for one-way Anova and Kruskal–Wallis test. *MM* mild–moderate, *MS* moderate–severe, *SAPS* Simplified Acute Physiology Score, *BMI* body mass index, E_rs_ respiratory system elastance

Table 2 gives the quantitative CT scan variables obtained at 5 cmH_2_O PEEP stratified as quartiles of P_ET_CO_2_/PaCO_2_. As shown, the well-aerated tissue increased with the P_ET_CO_2_/PaCO_2_ ratio. The poorly inflated, non-aerated tissue, the inhomogeneity index [10] and recruitability all significantly decrease throughout the P_ET_CO_2_/PaCO_2_ quartiles. As shown in Table 3, the P_ET_CO_2_/PaCO_2_ ratio was strongly associated with respiratory system elastance, alveolar ventilation and VCO_2_. These variables improved when the P_ET_CO_2_/PaCO_2_ ratio approached unity. The gas exchange variables under the same conditions are presented in Table 4. As shown, the PaCO_2_ progressively decreased with the concurrent increase of P_ET_CO_2_/PaCO_2_ ratio, while the PaO_2_/FiO_2_ ratio and saturation increased. Both venous admixture and dead space significantly decreased throughout the P_ET_CO2/PaCO_2_ quartiles.

**Table 2:** Computed tomography quantitative variables at 5 cmH_2_O of end-expiratory pressure among the P_ET_CO_2_/PaCO_2_ ratio quartiles

P_ET_CO_2_/PaCO_2_ quartiles	I	II	III	IV	*p* value
Ranges	[0.416,0.709]	[0.709,0.796]	[0.796,0.886]	[0.886,1.16]	
Number of patients	*N* = 52	*N* = 51	*N* = 48	*N* = 49	
Not aerated lung tissue, g	864 [534; 1321]	570 [417; 824]	510 [343; 692]	459 [314; 664]	< 0.001
Poorly aerated lung tissue, g	545 [342; 761]	398 [271; 552]	423 [284; 584]	356 [264; 440]	0.001
Normally aerated lung tissue, g	281 [189; 414]	288 [220; 452]	363 [243; 487]	356 [260; 500]	0.021
Hyperinflated lung tissue, g	0.32 [0.02; 2.72]	0.16 [0.01; 1.10]	0.24 [0.02; 2.47]	0.19 [0.01; 1.05]	0.739
Inhomogeneity index	0.20 [0.15; 0.24]	0.16 [0.13; 0.23]	0.15 [0.12; 0.18]	0.14 [0.12; 0.20]	0.007
Possibly recruitable tissue, g	336 [168; 583]	253 [109; 462]	171 [89.8; 295]	117 [54.4; 219]	< 0.001

Data are expressed in medians and IQR. *P* for Mann–Whitney test

**Table 3:** Respiratory mechanics and hemodynamics across P_ET_CO_2_/PaCO_2_ ratio quartiles

P_ET_CO_2_/PaCO_2_ quartiles	I	II	III	IV	*p* value
Range	[0.416,0.709]	(0.709,0.796]	(0.796,0.886]	(0.886,1.16]	
Number of patients	*N* = 52	*N* = 51	*N* = 48	*N* = 49	
*Respiratory variables*					
*E*_rs,_ cmH_2_O/L	27.9 [21.4; 33.5]	23.6 [20.4; 30.0]	25.7 [20.1; 30.1]	22.3 [18.1; 27.4]	0.015
Tidal volume, mL	480 [420; 550]	520 [455; 598]	510 [451; 600]	540 [485; 598]	0.064
RR, bpm	18.0 [14.8; 20.0]	16.0 [13.0; 19.0]	16.0 [13.0; 18.0]	15.0 [14.0; 18.0]	0.414
MAP, cmH_2_O	12.0 [10.0; 13.0]	11.0 [9.00; 13.0]	11.0 [10.0; 12.0]	10.0 [9.83; 11.5]	0.130
*V*_A_ L/min	3.18 [2.74; 4.75]	3.99 [2.86; 5.12]	4.25 [3.44; 5.60]	4.72 [3.33; 5.74]	0.003
VCO_2_, mL/min	117 [81.2; 137]	144 [112; 174]	164 [129; 189]	180 [144; 200]	< 0.001
*Hemodynamics*					
HR, bpm	87.0 [71.0; 105]	89.0 [70.5; 96.0]	89.0 [81.0; 106]	96.0 [82.0; 108]	0.245
Mean arterial pressure, mmHg	77.0 [69.5; 86.7]	83.0 [73.7; 90.3]	79.0 [72.0; 87.5]	81.7 [73.2; 89.2]	0.210
CVP, mmHg	11.0 [9.00; 14.0]	11.0 [9.00; 13.0]	12.0 [9.50; 14.0]	12.0 [9.00; 14.0]	0.822

Variables are expressed in medians and IQR. *P* for Mann–Whitney test. *E*_*rs*_ respiratory system elastance, *RR* respiratory rate, *PEEP* end-expiratory pressure, *MAP* mean airway pressure, *V*_A_ alveolar ventilation, *HR* heart rate, *CVP* central venous pressure

**Table 4:** Gas exchange across P_ET_CO_2_/PaCO_2_ ratio quartiles

P_ET_CO_2_/PaCO_2_ quartiles	I	II	III	IV	*p* value
Ranges	[0.416,0.709]	[0.709,0.796]	[0.796,0.886]	[0.886,1.16]	
Number of patients	*N* = 52	*N* = 51	*N* = 48	*N* = 49	
PaO_2,_ mmHg	62.6 [55.0; 73.6]	71.0 [63.5; 81.2]	75.0 [65.8; 89.7]	71.8 [65.0; 83.0]	0.001
PaCO_2,_ mmHg	48.1 [43.3; 53.8]	46.0 [41.1; 52.4]	44.7 [40.4; 50.1]	39.9 [36.5; 43.2]	< 0.001
PaO_2_/FiO_2_	103 [76.7; 131]	117 [102; 158]	164 [139; 203]	171 [143; 207]	< 0.001
Arterial pH	7.36 (0.07)	7.37 (0.07)	7.38 (0.08)	7.40 (0.07)	0.009
SaO_2_	90.8 [87.5; 94.0]	92.4 [90.5; 95.3]	94.1 [92.0; 96.2]	94.7 [92.7; 96.8]	< 0.001
Venous pH	7.34 (0.07)	7.35 (0.06)	7.36 (0.07)	7.37 (0.05)	0.141
PvO_2,_ mmHg	42.1 [39.8; 45.6]	42.0 [39.2; 44.1]	40.8 [37.2; 49.0]	42.9 [40.0; 47.7]	0.667
PvCO_2,_ mmHg	50.2 [46.9; 56.9]	52.0 [46.8; 55.3]	49.8 [45.0; 56.5]	45.8 [41.0; 47.3]	< 0.001
SvO_2_	73.5 [70.1; 76.5]	74.3 [71.4; 77.7]	76.4 [70.0; 80.6]	77.7 [72.6; 81.0]	0.073
*v*-*a* difference	2.31 [1.30; 2.83]	2.46 [1.92; 2.83]	2.65 [2.00; 3.26]	2.10 [1.56; 2.53]	0.036
*Q*_s_/*Q*_t_	0.54 [0.40; 0.66]	0.42 [0.36; 0.48]	0.35 [0.27; 0.43]	0.39 [0.30; 0.46]	< 0.001
*V*_d_/*V*_t_	0.74 [0.67; 0.80]	0.63 [0.60; 0.68]	0.56 [0.51; 0.61]	0.47 [0.44; 0.55]	< 0.001

Variables are expressed in medians and IQR or means ± SD according to distribution. *P* for Mann–Whitney test or one-way Anova. *Q*_s_/*Q*_t_: shunt fraction; *V*_d_/*V*_t_: dead space fraction

### P_ET_CO_2_/PaCO_2_ ratio at different airway pressures

**Fig. 3:**
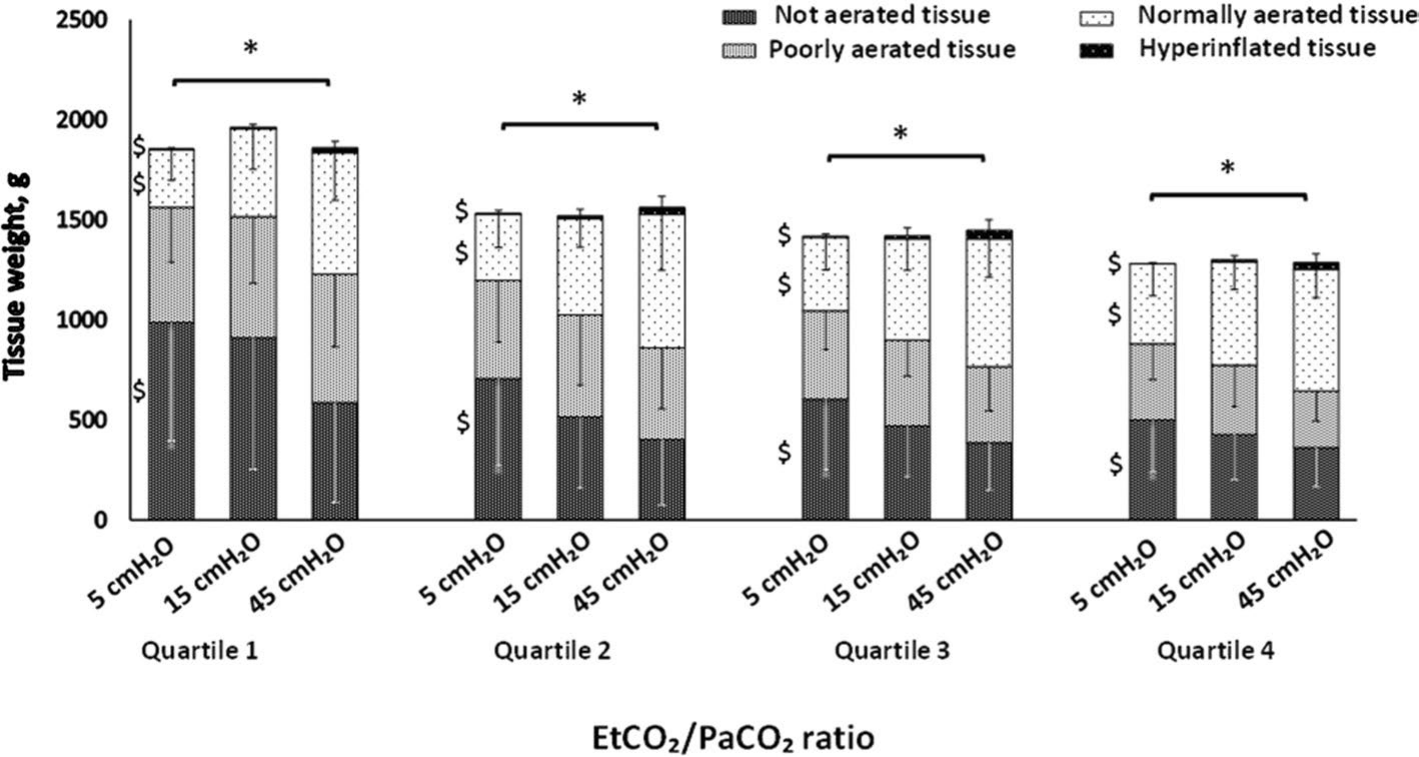
Portions of lung tissue classified as not aerated, poorly aerated, normally aerated and hyperinflated among P_ET_CO_2_/PaCO_2_ ratio quartiles to the response of step increase of PEEP at 5, 15 and 45 cmH_2_O. The asterisk denotes *p* < 0.001 among the portions of tissue throughout P_ET_CO_2_/PaCO_2_ ratio quartiles for Mann–Whitney test. The dollar denotes *p* < 0.001 among portions of lung tissue to the response to PEEP for Wilcoxon test

Figure 3 illustrates how lung tissue aeration changed in the different P_ET_CO_2_/PaCO_2_ ratio quartiles when airway pressure was increased from 5 to 15 and 45 cmH_2_O. The amount of non-aerated tissue decreased steadily from 5 to 45 cmH_2_O in all quartiles, while the amount of normally aerated tissue increased.

In Table 5, we report the changes of CT scan, respiratory mechanics and gas exchange variables through the quartiles of P_ET_CO_2_/PaCO_2_ when PEEP was increased from 5 to 15 cmH_2_O. As shown, the greatest improvement of non-aerated tissue, PaO_2_ and venous admixture were observed in quartile 1 of P_ET_CO_2_/PaCO_2_ and the worst deterioration of dead space in quartile 4.

**Table 5:** Changes in CT scan, respiratory mechanics and gas exchange variables in response to PEEP increase from 5 to 15 cmH_2_O among P_ET_CO_2_/PaCO_2_ ratio quartiles

P_ET_CO_2_/PaCO_2_quartiles Δ	I	II	III	IV	*p* value
Ranges	[0.416,0.709]	[0.709,0.796]	[0.796,0.886]	[0.886,1.16]	
Number of patients	*N* = 52	*N* = 51	*N* = 48	*N* = 49	
Δ Lung volume, mL	579 [417; 775]	602 [436; 719]	588 [467; 735]	586 [443; 823]	0.98
Δ Lung gas, mL	546 [451; 816]	598 [413; 711]	551 [456; 700]	564 [395; 810]	0.99
Δ Lung tissue, g	10.9 [− 42; 53]	26 [− 5.1; 53]	20 [6.4; 43.4]	22.6 [− 23; 56]	0.87
Δ Not aerated tissue, mL	− 170 [− 282; − 78]	− 87.7 [− 160; − 42.3]	− 70.1 [− 124.4; − 40.3]	− 23.5 [− 91.5; 0.3]	< 0.001
Δ Poorly aerated tissue, mL	7.3 [− 50; 171]	− 42.4 [− 97.1; 14.1]	− 37.2 [− 105.7; 15]	− 73 [− 112; − 17]	0.02
Δ Normally aerated tissue, mL	140 [82; 241]	153 [68; 226]	143 [94; 197]	134 [87; 199]	0.96
Δ Hyperinflated tissue, mL	1.5 [0.17; 4]	0.85 [0.13; 1.8]	0.5 [0.04; 3.44]	0.5 [0.11; 5.4]	0.92
Δ *Q*_s_/*Q*_t_	− 0.13 [− 0.2; − 0.08]	− 0.08 [− 0.13; − 0.04]	− 0.08 [− 0.14; − 0.01]	− 0.07 [− 0.13; − 0.03]	0.01
Δ *V*_d_/*V*_t_	0.01 [0.0; 0.03]	0.02 [− 0.01; 0.04]	0.01 [0.0; 0.04]	0.03 [0.02; 0.06]	0.03
Δ PaO_2_, mmHg	32 [18.5; 46]	23 [10.2; 58.4]	21.2 [4.3; 36]	16 [4.4; 28]	0.01
Δ *E*_RS_, cmH_2_O/L	0.07 [− 4.0; 3.4]	− 0.2 [− 2.2; 4.1]	0.3 [− 4.1; 2.4]	0.0 [− 2.5; 2.2]	0.85

Differences were computed as the variable at PEEP 15 cmH_2_O minus the variable at PEEP 5 cmH_2_O. Variables are expressed in medians and IQR. *P* for Mann–Whitney test. *Q*_s_/*Q*_t_: shunt fraction; *V*_d_/*V*_t_: dead space fraction; *P*/*F*: PaO_2_/FiO_2_ ratio; *E*_RS_: respiratory system elastance; SvO_2_: central venous oxygen saturation; P_ET_CO_2_: end-tidal CO_2_

## Discussion

In this study, we found that the P_ET_CO_2_/PaCO_2_ ratio is strongly associated with most of the morphological and physiological characteristics of ARDS, resulting as an easy and appealing measure of the status and the performance of the lung.

**Fig. 4:**
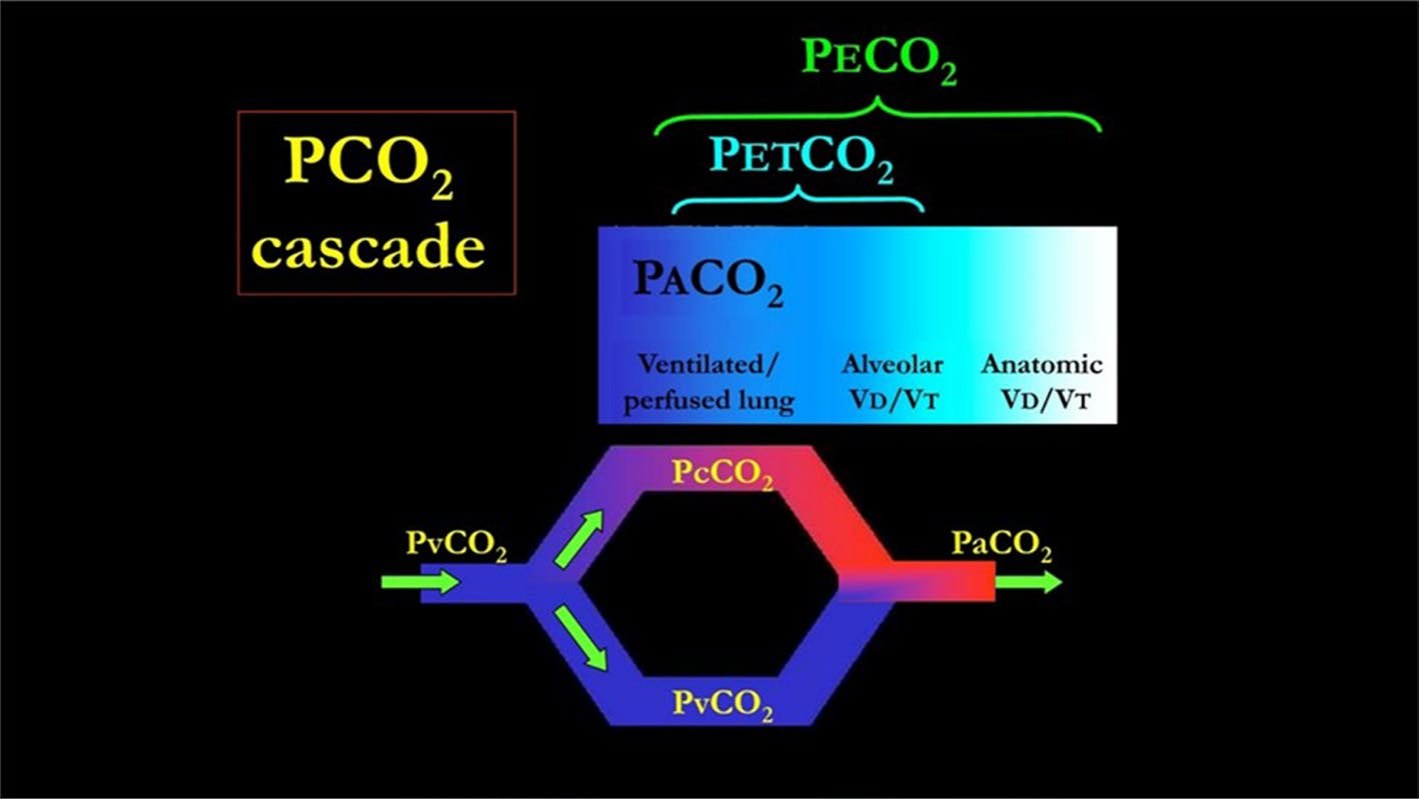
The PvCO_2_ decreases to PcCO_2_ (the capillary PCO_2_ pressure) after releasing CO_2_ in the alveolar space. The partial pressure of CO_2_ in the alveoli (PACO_2_) which are perfused is equal to PcCO_2_. This pressure represents the “ideal” alveolar gas, as it derives from pulmonary units which are both perfused and ventilated. This model ignores other factors possibly affecting the CO_2_ dynamics, such as the CO_2_ production from the inflammatory cells residing in the inflamed lung. Actually, this ideal PACO_2_ may be “diluted” by the gas coming from unperfused but ventilated alveoli (alveolar dead space). The PACO_2_ then becomes P_ET_CO_2_. In turn, the P_ET_CO_2_ may be diluted into the gas ventilating the airways and the apparatus (anatomical dead space) becoming P_ET_CO_2_. It must be realized that the number of molecules of CO_2_ involved are exactly the same, but their concentration (and partial pressure) depends on the progressive dilution with alveolar gases and “anatomical” gases. In normal individual, PACO_2_ and P_ET_CO_2_ differ only of 1–2 mmHg, being the alveolar dead space negligible. The PvCO_2_ may also reach the arterial circulation without any modification in presence of venous admixture. In this case, PaCO_2_ is the result of mixing between PcCO_2_ and PvCO_2_. Greater is the venous admixture, greater is the difference between PaCO_2_ (higher) and PcCO_2_. In the perfect gas exchanger, P_ET_CO_2_ equals the PACO_2_ (no alveolar dead space) and PACO_2_ equals PaCO2 (no venous admixture). Therefore P_ET_CO_2_/PaCO_2_ equals 1

The physiological meaning of the P_ET_CO_2_/PaCO_2_ ratio may be easily understood when one considers CO_2_ kinetics through the anatomical space from the pulmonary capillaries to the airway opening. Figure 4 shows that, in the ideal lung, PaCO_2_ is equal to PcCO_2_ (venous admixture fraction = 0). Similarly, P_ET_CO_2_ is equal to PACO_2_ (alveolar dead space fraction = 0). Therefore, in this “ideal” setting the ratio of P_ET_CO_2_ to PaCO_2_ would be 1. This ratio will depart progressively from 1 in the presence of a venous admixture and/or alveolar dead space. Consequently, the P_ET_CO_2_/PaCO_2_ ratio is a rather unspecific variable, as it is linked to both CO_2_ and O_2_ exchange impairment, but for the same reason it may give an immediate warning of an overall impairment of gas exchange. The potential role of monitoring the P_ET_CO_2_/PaCO_2_ ratio in order to follow and understand the disease course is emphasized by its close association with the overall severity of ARDS and the mortality.

This is not really surprising, as almost all variables characterizing the ARDS are related to the P_ET_CO_2_/PaCO_2_ ratio. With regard to the morphological variables, the P_ET_CO_2_/PaCO_2_ ratio is related to the extent of non-aerated and aerated tissue, the size of the baby lung as well as to the extent of recruitability. No other gas exchange variable exhibits such a large number of correlations with lung morphology. Indeed, venous admixture and physiological dead space were only related to the non-aerated tissue and to the normally aerated tissues, respectively. Therefore, the relative nonspecificity of the P_ET_CO_2_/PaCO_2_ ratio, which reflects the overall gas exchange (both for oxygen and carbon dioxide), explains its correlation with all the morphological components of the lungs, of which some are more related to oxygen while others are more related to CO_2_ exchange. On the other hand, the linkage between the P_ET_CO_2_/PaCO_2_ ratio, alveolar dead space and venous admixture accounts for its high sensitivity in detecting an overall impairment in gas exchange. The P_ET_CO_2_/PaCO_2_ ratio was inversely associated with respiratory system elastance and directly correlated with alveolar ventilation, while no relationship was found with hemodynamic variables. The low specificity but high sensitivity of the P_ET_CO_2_/PaCO_2_ ratio to reflect an overall impairment of gas exchange is strikingly shown by the correlation we found with the gas exchange variables. Indeed, as shown in Table 4, all measured or computed variables related either with oxygenation or carbon dioxide clearance were strongly associated with the P_ET_CO_2_/PaCO_2_ ratio. Therefore, an altered P_ET_CO_2_/PaCO_2_ ratio, as such, is associated both with the morphology and, the function of gas exchange, suggesting it as a sensitive, easily available marker, of changes in lung conditions. Interestingly, we found that the VCO_2_ significantly increased throughout the quartiles. We believe that this is due to an improved alveolar ventilation, with greater elimination of carbon dioxide. Indeed, the low VCO_2_ measured in quartile 1 possibly represents only a fraction of the metabolic CO_2_ production which is partly retained. Increased alveolar ventilation throughout the quartiles leads to a normalization or even a higher than normal (metabolic) CO_2_ clearance [12].

The P_ET_CO_2_/PaCO_2_ ratio may be also considered to anticipate the PEEP response. The PEEP response in gas exchange is a balance between the decrease of venous admixture and increase in alveolar dead space. The venous admixture decrease could either be due to recruitment, better mechanical conditions of pulmonary units already open, or to a decrease in cardiac output, while the alveolar dead space increase may be due to an overdistention of pulmonary units relative to their perfusion. An increase in alveolar dead space would tend to reduce P_ET_CO_2_, while a decrease of right-to-left venous admixture would tend to reduce PaCO_2_. The P_ET_CO_2_/PaCO_2_ ratio is related to the two variables, and its changes may reflect these physiopathological mechanisms. Moreover, we showed that patients starting with a lower P_ET_CO_2_/PaCO_2_ ratio had a more favorable response to PEEP.

The P_ET_CO_2_/PaCO_2_ ratio is obviously related to the physiological dead space, although it may be considered a “positive variable” (greater the ratio, better the gas exchange) rather than “negative” (greater the dead space, worse the gas exchange). Indeed, any change of the P_ET_CO_2_/PaCO_2_ ratio toward the value of 1 indicates an improvement of the whole gas exchanger condition, which may then be easily monitored after any maneuvre on the respiratory system, change of ventilator setting and pharmacological intervention. Indeed, the daily monitoring of this easy to use variable may show a progressive increase towards the unity, giving some evidence that the lung conditions are improving. In contrast, any change of this ratio towards lower values immediately indicates an overall decrease of the gas exchange performances. The P_ET_CO_2_/PaCO_2_ ratio also helps the clinician to be more aware of the dynamics of CO_2_. Too often oxygenation represents the concern at the bedside of an ARDS patient, but it is only considering also PaCO_2_ that one can have a more complete picture of the lung structure and function [13]. Finally, the P_ET_CO_2_/PaCO_2_ ratio finds in its strength as an overall meter of gas exchange impairment also its weakness. Indeed, to fully understand the various components of the of the gas exchange alteration, both dead space and venous admixture must be be measured.

## Conclusions

In this study, we evaluated the P_ET_CO_2_/PaCO_2_ ratio as a clinical tool to comprehensively evaluate the gas exchange lung function. The ratio was associated with most of the physiological variables that can be measured at the bedside and, therefore, it can represent a useful parameter for the daily monitoring of the ARDS patient.

## Supplementary Information

**Additional file 1.** Additional methods and formulas.

## Data Availability

Dataset available upon reasonable request.
