# Supplementary material for: End-tidal to arterial PCO2 ratio: a bedside meter of the overall gas exchanger performance
Source: Intensive Care Med Exp. 2021 Apr 19;9:21. doi: 10.1186/s40635-021-00377-9 (PMC8054233; doi:10.1186/s40635-021-00377-9)
Supplement: Supplementary file 1 — Additional file 1. Additional methods and formulas. [file 40635_2021_377_MOESM1_ESM.docx]

# End-tidal to arterial PCO_2_ ratio: a bedside meter of the overall gas exchanger performance

Matteo Bonifazi^1^, Federica Romitti^1^, Mattia Busana^1^, Maria Michela Palumbo^1^, Irene Steinberg^1^, Simone Gattarello^1^, Paola Palermo^1^, Leif Saager^1^, Konrad Meissner^1^, Michael Quintel^1^, Davide Chiumello^2^, Luciano Gattinoni^1^

# ONLINE DATA SUPPLEMENT

**Additional Methods**

*Venous admixture*

Venous admixture was calculated according to the following equation:

$$\frac{Q_{s}}{Q_{t}}=\frac{{Cc}_{O2}- {Ca}_{O2}}{{Cc}_{O2}- {Cv}_{O2}}$$

Where

$Q_{s}$= Blood flow through shunt

$Q_{t}$= Total cardiac output

${Cc}_{O2}$= Oxygen content of pulmonary end-capillary blood

${Ca}_{O2}$= Oxygen content of arterial blood

${Cv}_{O2}$= Oxygen content of central mixed venous blood

*Physiological dead space fraction*

Physiological dead space fraction (V_d_/V_t_) was calculated according to the Bohr´s equation with Enghoff's modification:

$$\frac{V_{d}}{V_{t}}=\frac{{Pa}_{CO2}- {PE}_{CO2}}{{Pa}_{CO2}}$$

Where:

$V_{d}$ = physiological dead space volume

$V_{t}$ = tidal volume

${Pa}_{CO2}$ = partial pressure of carbon dioxide in the arterial blood

${PE}_{CO2}$ = partial pressure of carbon dioxide in the average expired (exhaled) air

*Alveolar dead space fraction*

Alveolar dead space fraction was calculated according to the Bohr´s following equation:

$$Alveolar dead space fraction=\frac{{Pa}_{CO2}- {P_{ET}}_{CO2}}{{Pa}_{CO2}}$$

Where:

${Pa}_{CO2}$ = partial pressure of carbon dioxide in the arterial blood

${PET}_{CO2}$ = partial pressure of carbon dioxide that is released at the end of an exhaled breath

Assuming that, P_ET_CO_2_ is representative of the alveolar gases, it follows that:

$${VA}_{TOT}\cdot P_{ET}{CO}_{2}={VCO}_{2}$$

If we define as “ideal” alveolar ventilation (VA_id_) the amount of the one which is perfused it follows that:

$${VA}_{id}\cdot{PACO}_{2}={VCO}_{2}$$

Assuming that the PaCO_2_ is equal to the PACO_2_ (true only when shunt = 0), from equation 1 and 2 it follows that:

$$\frac{{VA}_{id}}{{VA}_{tot}}=\frac{P_{ET}{CO}_{2}}{P_{a}{CO}_{2}}$$

Therefore:

$$Alveolar dead space= \frac{{VA}_{TOT}-{VA}_{id}}{{VA}_{TOT}}= \frac{{Pa}_{CO2}- {P_{ET}}_{CO2}}{{Pa}_{CO2}}$$
